# Analysis of risk factors for the development of cognitive dysfunction in patients with cerebral small vessel disease and the construction of a predictive model

**DOI:** 10.3389/fneur.2022.944205

**Published:** 2022-08-11

**Authors:** Le Zhang, Fulin Gao, Yamin Zhang, Pengjuan Hu, Yuping Yao, Qingzhen Zhang, Yan He, Qianlan Shang, Yi Zhang

**Affiliations:** ^1^The First Clinical Medical College of Gansu University of Chinese Medicine (Gansu Provincial Hospital), Lanzhou, China; ^2^The Department of Neurology, Gansu Provincial Hospital, Lanzhou, China

**Keywords:** cerebral small vessel disease, vascular dementia, cognitive dysfunction, risk factor, nomogram, predictive model

## Abstract

**Background:**

Cognitive dysfunction in cerebral small vessel disease (CSVD) is a common cause of vascular dementia. The purpose of this study was to find independent risk factors for the development of cognitive dysfunction in patients with CSVD and establish a risk prediction model, in order to provide a reference for clinical diagnosis and treatment of such patients.

**Methods:**

In this study, clinical data of patients with CSVD admitted to the Department of Neurology in Gansu Provincial Hospital from December 2019 to December 2021 were collected, and 159 patients were finally included after strict screening according to the inclusion and exclusion criteria. There were 43 patients with normal function and 116 patients with cerebral small vessel disease cognitive impairment (CSVDCI). The logistic multivariable regression model was used to screen out the independent risk factors of cognitive dysfunction in patients with CSVD, and the nomogram of cognitive dysfunction in patients with CSVD was constructed based on the results of the logistic multivariable regression analysis. Finally, the accuracy of the prediction model was evaluated by C-index, calibration curve, receiver operating characteristic (ROC) curve, and decision curve analysis (DCA).

**Results:**

The results of multivariable logistic regression analysis showed that hypertension (OR = 2.683, 95% CI 1.119–6.432, *P* = 0.027), homocysteine (Hcy) (OR = 1.083, 95% CI 1.026–1.143, *P* = 0.004), total CSVD MRI Score (OR = 1.593, 95% CI 1.025–2.475, *P* = 0.039) and years of schooling (OR = 0.883, 95% CI 0.798–0.978, *P* = 0.017) were independent risk factors for the development of cognitive dysfunction in patients with CSVD. The C-index of this prediction model was 0.806 (95% CI 0.735–0.877), and the calibration curve, ROC curve, and DCA curve all showed good predictive power in the nomogram.

**Conclusions:**

The nomogram constructed in this study has high accuracy and clinical utility in predicting the occurrence of cognitive dysfunction in patients with CSVD. For patients with CSVD with the above risk factors, active clinical intervention and prevention are required during clinical consultation and disease management to avoid cognitive impairment as much as possible.

## Introduction

Vascular cognitive impairment is one of the common causes of dementia in patients, of which cognitive dysfunction in CSVD accounts for a large proportion (1, 2). CSVD is a group of pathological processes affecting the penetrating arteries, microarteries, capillaries, small veins, and microvenules of the brain due to one or more etiologies, which in turn leads to brain parenchymal damage, and approximately 45% of cognitive decline and overall life loss (3–7). It has a great impact on the quality of life of patients. According to the etiology, CSVD includes six major categories, (5) including type 1:arteriolosclerosis (or age-related and vascular risk-factor-related small vessel diseases), type 2: sporadic and hereditary cerebral amyloid angiopathy, type 3: inherited or genetic small vessel diseases distinct from cerebral amyloid angiopathy, type 4: inflammatory and immunologically mediated small vessel diseases, type 5: venous collagenosis, and type 6: other small vessel diseases. Among them, type 1 and type 2 (6) are the most common types, and this study focuses on type 1 (3).

At present, the pathogenesis of the disease is still unclear, and some studies have found that age, living habits, and risk factors related to cerebrovascular disease may affect the cognitive dysfunction of patients with CSVD (3, 8–10). However, the results of risk factors were not consistent, and there was no nomogram for establishing predictive models based on relevant risk factors. This study expects to enrich the necessary findings. As a new type of statistical prediction model, nomogram can convert risk factors into a graph of a continuous scoring system. Compared with traditional predictive analysis models, nomogram has many advantages such as high accuracy, flexibility in use, and ease of generalization (11–13). Nomograms can be used clinically to predict risk, to help physicians make personalized treatment decisions and to be more easily understood and cooperated with by patients in doctor-patient communication, and are now used in a variety of clinical studies. This study aimed to find possible risk factors for the development of cognitive dysfunction in CSVD patients and to construct a predictive model for the visual presentation of the nomogram, in order to provide a reference for clinicians in the assessment, disease management, and clinical interventions for patients with CSVD.

## Methods

### Patients and selection criteria

A total of 159 patients with CSVD admitted to the Department of Neurology in Gansu Provincial Hospital from December 2019 to December 2021 were selected, and the clinical data of these patients were statistically analyzed. Among them, 43 patients with normal cognitive function in CSVD and 116 patients with CSVDCI. The inclusion criteria were as follows: (1) the diagnoses of the patients were all in accordance with the Chinese Consensus on the Diagnosis and Management of Cerebral Small Vessel Disease (14); (2) cranial magnetic resonance imaging (MRI) examination showed one or more of the following imaging features: lacunar infarction (LI), white matter hyperintensity (WMH), enlarged perivascular space (ePVS), and cerebral microbleeds (CMBs); (3) age ≥ 50 years; (4) only patients with small atherosclerotic CSVD were included (15); (5) no history of stroke or dementia; (6) not taking any medications that affect cognitive function, such as anticholinergics, sedative-hypnotics, antihistamines, etc.; (7) patients and their families agreed and voluntarily joined this study. The exclusion criteria were as follows: (1) patients with contraindications to MRI examination; (2) Imaging findings of intracranial occupying lesions such as brain tumors, hydrocephalus, or non-cerebral small vessel white matter lesions (e.g., multiple sclerosis, tuberous disease, brain radiation therapy); (3) patients with other central nervous system lesions or severe medical diseases; (4) patients with a history of depression and bipolar disorder; (5) patients with a history of COVID-19; (6) patients with congenital mental retardation and dementia caused by other diseases (such as severe mental illness, epilepsy, alcohol and drug abuse, poisoning, etc.).

### Study variables

Patients' age, sex, ethnicity, BMI, years of schooling, smoking (defined as smoking ≥1 cigarette per day on average and smoking history ≥1 year), drinking (defined as drinking ≥50 ml of liquor on average per day, ≥1 time per week on average and drinking history ≥1year), hypertension, coronary heart disease, diabetes, systolic blood pressure (in the early morning of the second day of admission to the hospital), triglycerides (TG), total cholesterol (TC), low-density lipoprotein (LDL), homocysteine (Hcy), C-reactive protein (CRP), blood urea nitrogen (BUN), creatinine (Cr), and carotid atheromatous plaque were collected as clinical factors.

### Cognitive function assessment

Cognitive assessment was based on the MOCA scale (16, 17), which includes eight cognitive domains including abstract thinking, attention and concentration, executive function, language, memory, visual structure skills, computation, and orientation. A MOCA total score of 26–30 indicates normal cognitive function, and a total score of <26 indicates cognitive dysfunction. If the education years ≤ 12, 1 point will be added.

### The total CSVD MRI score

MRI images were blindly reviewed by two experienced neurologists with reference to the STRIVE criteria (18), and a total CSVD MRI score (8) was used to reflect CSVD severity: 1 point for LI ≥1; 1 point for CMBs ≥1; 1 point for moderate to severe ePVS (grade 2–4); Fazekas graded 3 points for periventricular white matter hyperintensity or ≥2 points for deep brain white matter hyperintensity, 1 point. The total score is 0–4 points, and the degree is graded: mild (0–1 points), moderate to severe (2–4 points). The scoring criteria for WMH, LI, CMBs, and ePVS are shown in Table 1.

**Table 1:** WMH, LI, CMBs, and ePVS scoring criteria for imaging examinations.

**WMH**	High signal in the lateral paraventricular or deep brain white matter on T2WI or FLAIR sequences and iso/low signal on T1WI, which may vary in magnitude, with the severity assessed by Fazekas classification				
	Periventricular white matter hyperintensities (PVWMHs)	0 points	1 point	2 points	3 points
		No	Caps or pencil lining	Smooth halo	Irregular periventricular hyperintensity extending into deep white matter
	Deep white matter hyperintensities (DWMHs)	0 points	1 point	2 points	3 points
		No	Punctuate lesions	Beginning confluence of lesions	Large confluent lesions
LI	A central cerebrospinal fluid-like low signal on T2WI and FLAIR sequences, surrounded by high signal but not high signal on DWI, distributed in the subcortex, round or oval-like, 3–15 mm in diameter				
CMBs	Foci of signal deficits on SWI that are round or similarly round, homogeneous in texture, with a clear border of 2 to 5 mm in diameter, located in the subepisternal, deep and cerebral lobes				
ePVS	Interstitial fluid-filled signal interstitially encircling vessels penetrating in the gray or white matter, point-like or linear shadow with cerebrospinal fluid isosignal on T1WI, T2WI, and FLAIR sequences, usually <3 mm in diameter, without circumferential enhancement and occupancy effects, graded at the level with the highest number of unilateral basal ganglia ePVS				
	0	Grade 1	Grade 2	Grade 3	Grade 4
	No	1 to 10 perivascular spaces	11 to 20 perivascular spaces	21 to 40 perivascular spaces	>40 perivascular spaces or uncountable

### Statistical analysis

Data from this study were statistically analyzed using SPSS 26.0 and R software (version 4.1.2, https://www.r-project.org/). Numeric variables are expressed as χ ± s, and independent samples *t*-test was used when both samples were from normal overall and the overall variance was equal; count data are expressed as absolute values or rates. Categorical variables were analyzed using the χ^2^ test (Pearson chi-square, Continuous correction chi-squared test, Fisher's exact test), possible risk factors were analyzed using logistic univariate regression analysis, and clinical factors with differential results (*P* < 0.05) from logistic univariate regression analysis were further included in the logistic multivariable regression analysis. Logistic multivariable regression models were analyzed using stepwise regression (Forward: LR) to screen for independent risk factors and establish regression equations. Based on the screened independent risk factors, a nomogram of the prediction model was constructed using the R software package “rms.” The receiver operating characteristic (ROC) curve of the prediction model was plotted, and the discriminatory ability of the model was evaluated by the area under the curve (AUC). The accuracy of the model was evaluated by calculating the consistency index (C-index) and plotting the calibration curve. The AUC values and C-index are between 0.50–0.70 for low accuracy, between 0.71–0.90 for moderate accuracy, and above 0.90 for high accuracy. In addition, a decision curve analysis (DCA) of the model was developed using the “ggDCA” package to quantify the net benefit rate at different threshold probabilities to assess the clinical validity of the model. *P* < 0.05 was considered statistically different, and confidence intervals for the parameters were estimated at 95% confidence intervals.

## Results

### Comparison of baseline information between the two groups of patients

A total of 159 CSVD patients were included after strict screening according to the inclusion and exclusion criteria. Among them, 43 patients with normal cerebral small vessel disease cognitive function (CSVD) and 116 patients with cerebral small vessel disease cognitive function impairment (CSVDCI). There are 77 males and 82 females; 152 cases were Han Chinese and 7 cases were Hui or Dongxiang, and the patients' ages ranged from 50 to 88 years. The baseline information is shown in Table 2.

**Table 2:** Clinical baseline data of patients with cerebral small vessel disease.

**Clinical Factors**	**CSVDCI**	**CSVD**	***t*/χ^2^**	***P value***
	**(*n* = 116)**	**(*n* = 43)**		
Age (years)	72.57 ± 9.65	67.77 ± 10.11	−2.752	0.007
**Sex**			0.004	0.950
Male	56 (48.28%)	21 (48.84%)		
Female	60 (51.72%)	22 (51.16%)		
**Ethnicity**			1.470	0.225
Han	109 (93.97%)	43 (100%)		
Hui or Dongxiang	7 (6.03%)	0		
**Smoking**			0.202	0.653
Yes	9 (7.76%)	5 (11.63%)		
No	107 (92.24%)	38 (88.37%)		
**Drinking**			1.869	0.172
Yes	46 (39.66%)	12 (27.91%)		
No	70 (60.34%)	31 (72.09%)		
**Hypertension**			10.519	0.001
Yes	90 (77.59%)	22 (51.16%)		
No	26 (22.41%)	21 (48.84%)		
**Coronary heart disease**			0.719	0.396
Yes	7 (6.03%)	5 (11.63%)		
No	109 (93.97%)	38 (88.37%)		
**Diabetes**			0.908	0.341
Yes	30 (25.86%)	8 (18.60%)		
No	86 (74.14%)	35 (81.40%)		
Systolic pressure	137.78 ± 19.21	131.02 ± 15.81	−2.062	0.041
BMI	25.49 ± 22.23	24.26 ± 3.48	−0.361	0.718
Years of schooling	8.67 ± 4.78	11.30 ± 3.64	3.272	0.001
TG	2.02 ± 1.38	1.50 ± 0.67	−2.367	0.019
LDL	2.47 ± 0.84	2.46 ± 0.79	−0.110	0.912
BUN	6.67 ± 2.29	5.91 ± 1.52	−2.007	0.046
Cr	71.11 ± 21.58	70.49 ± 17.79	−0.169	0.866
Hcy	28.39 ± 30.17	14.98 ± 6.49	−2.884	0.004
CRP	9.11 ± 19.52	2.58 ± 4.07	−2.174	0.031
TC	4.59 ± 2.37	4.25 ± 0.99	−0.911	0.364
Total CSVD MRI Score	1.51 ± 1.09	0.77 ± 1.00	−3.891	<0.001
**Carotid atheromatous plaque**			12.121	<0.001
Yes	103 (88.79%)	28 (65.12%)		
No	13 (11.21%)	15 (34.88%)		

### Logistic univariate regression analysis results

The univariate logistic analysis of all clinical factors showed that age, systolic blood pressure, years of schooling, hypertension, TG, BUN, Hcy, CRP, total CSVD MRI Score, and carotid atheromatous plaque were factors associated with the development of cognitive dysfunction in patients with CSVD (*P* < 0.05). In contrast, sex, ethnicity, smoking, drinking, coronary heart disease, diabetes, BMI, LDL, Cr, and TC were not factors associated with the development of cognitive dysfunction in patients with CSVD (*P* ≥ 0.05). The results are shown in Table 3.

**Table 3:** Logistic univariate and multivariable regression analysis of cognitive impairment in patients with CSVD.

**Variables**	**Logistic univariate regression analysis**		**Logistic multivariable regression analysis**	
	**OR (95% CI)**	***P* value**	**OR (95% CI)**	***P* value**
Age (years)	1.048 (1.012–1.086)	0.009		
**Sex**				
Female	Reference			
Male	0.978 (0.485–1.969)	0.950		
**Ethnicity**				
Hui or Dongxiang	Reference			
Han	0.000 (0.000–NA)	0.999		
**Smoking**				
No	Reference			
Yes	0.639 (0.202–2.027)	0.447		
**Drinking**				
No	Reference			
Yes	1.698 (0.791–3.642)	0.174		
**Hypertension**				
No	Reference		Reference	
Yes	3.304 (1.576–6.927)	0.002	2.683 (1.119–6.432)	0.027
**Coronary heart disease**				
No	Reference			
Yes	0.488 (0.146–1.630)	0.244		
**Diabetes**				
No	Reference			
Yes	1.526 (0.637–3.655)	0.343		
Systolic pressure	1.021 (1.001–1.042)	0.043		
BMI	1.005 (0.979–1.030)	0.728		
Years of schooling	0.869 (0.796–0.950)	0.002	0.883 (0.798–0.978)	0.017
TG	1.660 (1.076–2.561)	0.022		
LDL	1.024 (0.670–1.566)	0.912		
BUN	1.241 (1.003–1.535)	0.047		
Cr	1.002 (0.984–1.019)	0.865		
Hcy	1.095 (1.038–1.156)	0.001	1.083 (1.026–1.143)	0.004
CRP	1.106 (1.027–1.191)	0.008		
TC	1.142 (0.856–1.523)	0.366		
Total CSVD MRI Score	2.059 (1.384–3.064)	<0.001	1.593 (1.025–2.475)	0.039
**Carotid atheromatous plaque**				
No	Reference			
Yes	4.245 (1.810–9.952)	0.001		

### Logistic multivariable regression analysis results

The clinical factors with significant (*P* < 0.05) univariate analysis results were further included in the logistic multivariable regression analysis, and the results showed that hypertension, years of schooling, Hcy, and total CSVD MRI Score were independent risk factors for the development of cognitive dysfunction in patients with CSVD (*P* < 0.05). Among them, high years of schooling was a protective factor, and hypertension, high Hcy, and high total CSVD MRI Sorce were risk factors. The results are shown in Table 3.

### Construction of the nomogram

**Figure 1:**
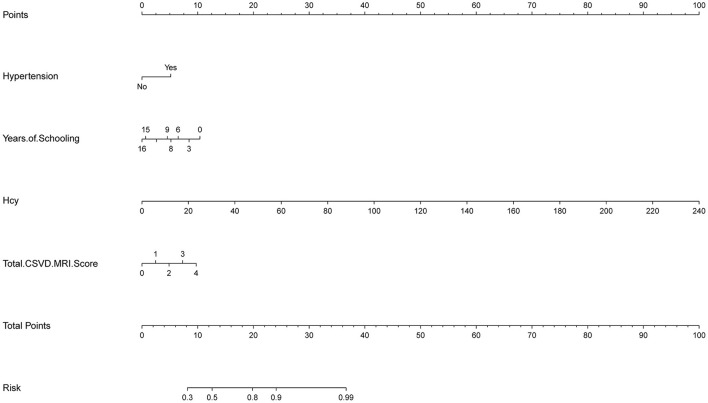
Nomogram of Hypertension, years of schooling, Hcy, and Total CSVD MRI Score.

In this study, based on the results of logistic multivariable regression analysis, four independent risk factors of cognitive impairment in CSVD patients were incorporated into the nomogram, and a nomogram was established to predict the risk of cognitive impairment in CSVD patients. For each patient, the total score was obtained by summing the scores of the corresponding variables, and the predicted probability of cognitive dysfunction for that patient was finally calculated by converting the relationship between the total score and the probability of occurrence of the outcome event as a function of the total score (Figure 1).

### Internal validation of nomogram and model performance

**Figure 2:**
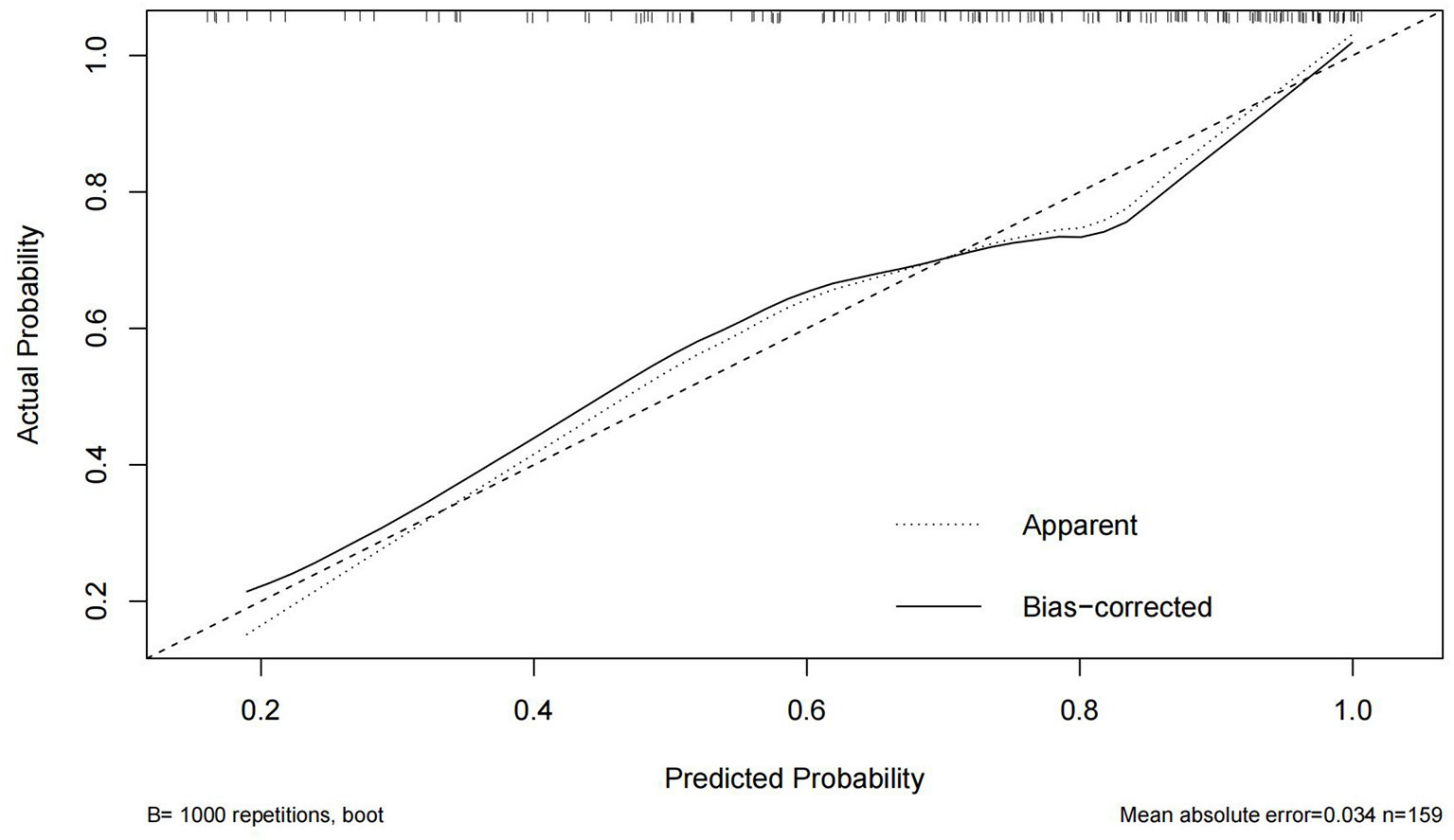
Calibration curve for internal validation of the nomogram.

In this study, the Bootstrap validation method was used to plot the calibration curve of the prediction model, the 45° line was used as the optimal model, and the results showed that the prediction results of the nomogram were in good agreement with the actual results, as shown in Figure 2. The C-index is 0.806 (95% CI 0.735–0.877), indicating that the model has good discriminatory ability and accuracy.

**Figure 3:**
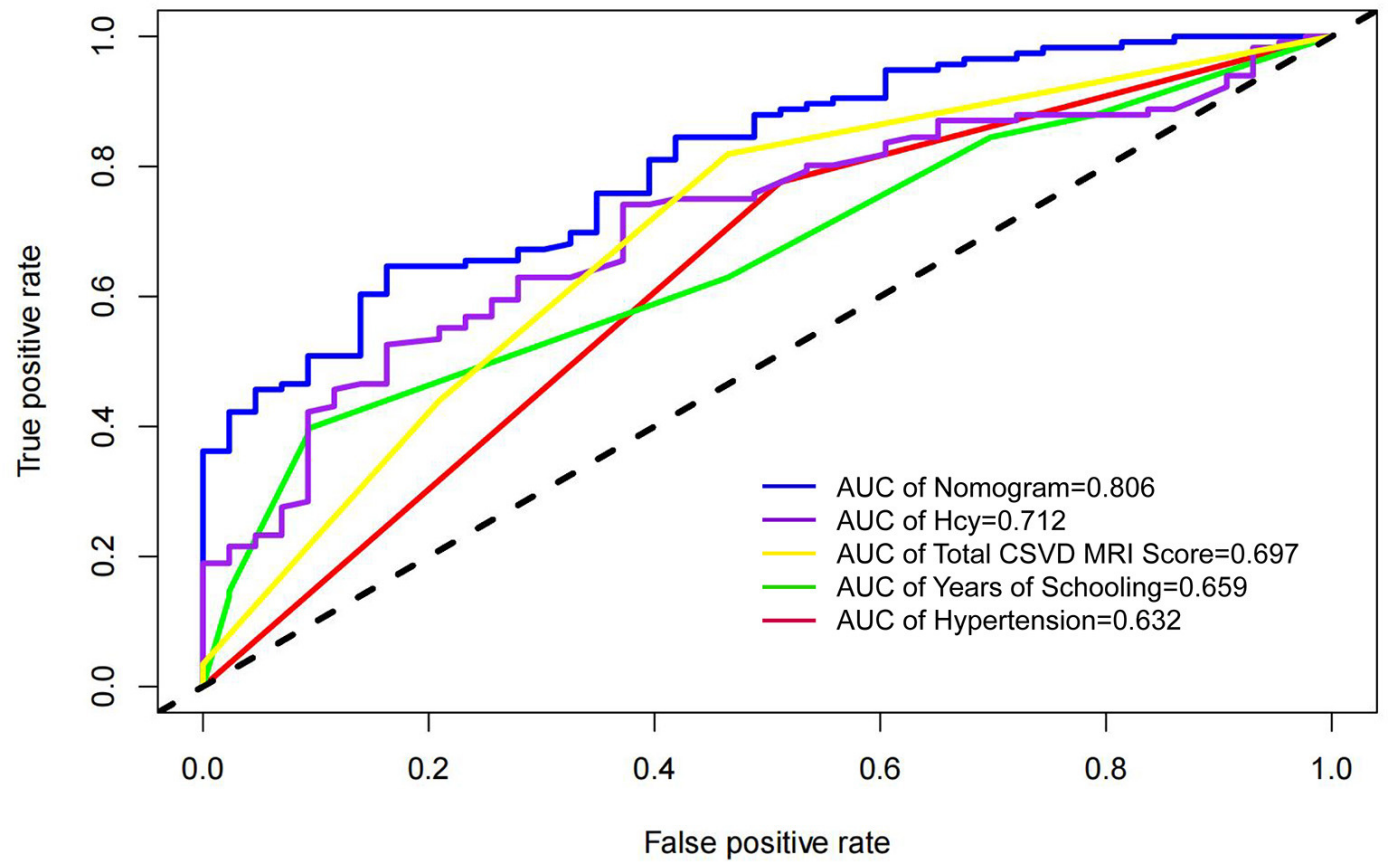
ROC curves of independent risk factors and nomogram.

In this study, four independent factors and the nomogram were used as measures in the ROC curve analysis (Figure 3). The results showed that the nomogram AUC value was 0.806, which was the largest, and the AUCs for hypertension, Hcy, total CSVD MRI Sorce, and years of schooling were 0.632, 0.712, 0.697, and 0.659, respectively, indicating that the predictive power of the nomogram is better than that of individual factors.

**Figure 4:**
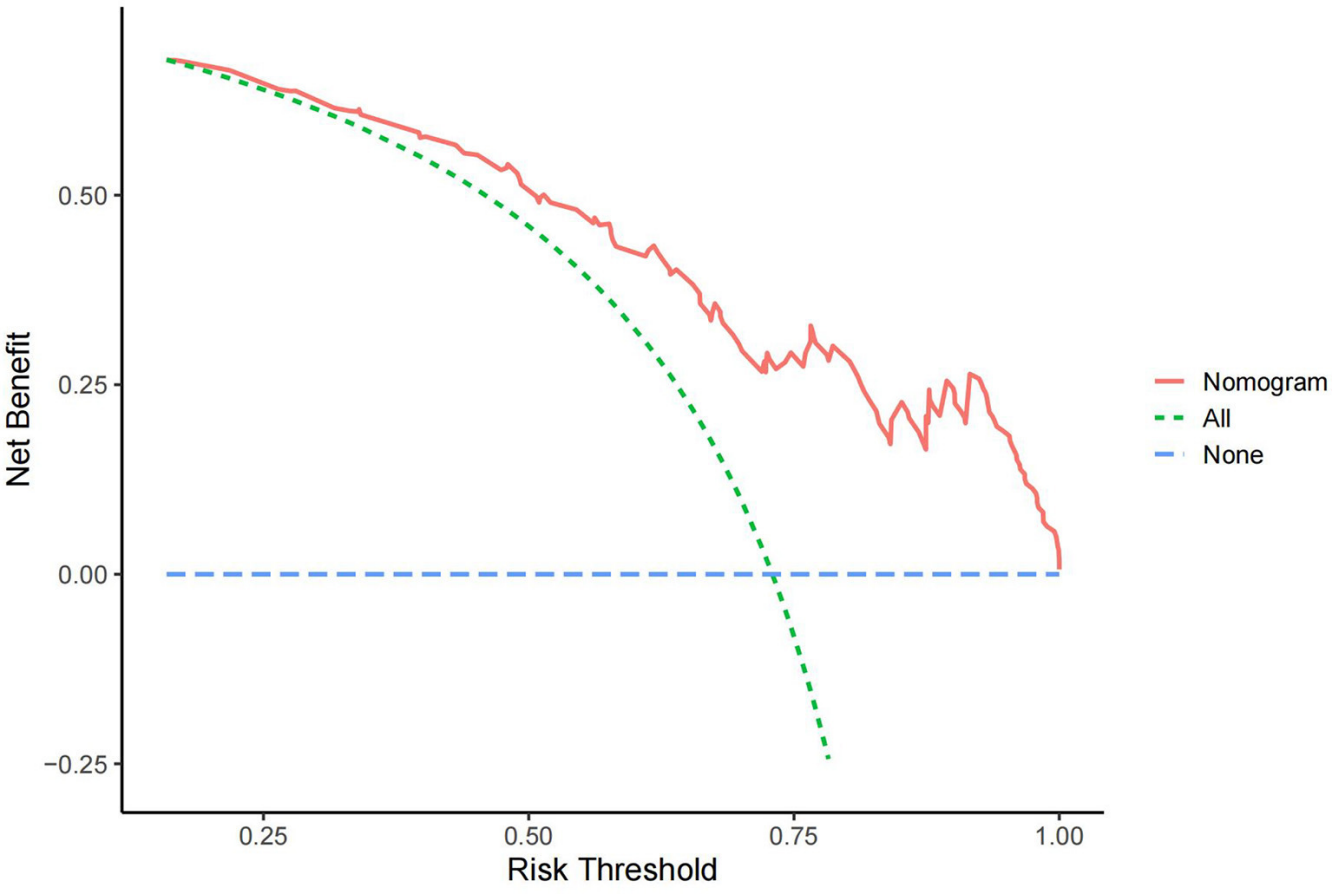
DCA curve of the nomogram.

In the DCA curve of the nomogram, the blue dotted line indicates a benefit rate of 0 assuming that all CSVD patients do not develop cognitive impairment and are left untreated; The green dotted line indicates that all CSVD patients developed cognitive impairment and all received treatment, with the net benefit being a backslash line with a negative slope. Within the threshold probability range of the DCA curves, patients had a higher net benefit than the other two extreme curves. The cut-off value obtained by ROC curve analysis of the nomogram (76.60%) was within the threshold probability range of the DCA curve, indicating the clinical validity of the model. Therefore, setting 76.60% as the threshold probability value for diagnosing cognitive dysfunction in patients with CSVD and treating patients with clinical interventions showed a good net benefit (Figure 4).

## Discussion

Cerebral small vessel disease (CSVD) is a common age-related cerebrovascular disease, and its pathological changes may be related to slowly accumulating tissue damage, which is one of the main causes of cognitive decline in the elderly (19). At present, the pathogenesis of cognitive dysfunction in CSVD remains unclear. It has been suggested that cognitive dysfunction in CSVD patients may be related to atherosclerosis and microvascular dysfunction (20, 21), which may affect cerebral blood perfusion, neurogenesis, and brain self-regulation, resulting in neurological dysfunction and cognitive dysfunction in CSVD patients (20–22). The purpose of this study was to investigate the independent risk factors for the development of cognitive dysfunction in patients with CSVD, with the aim of providing a reference for clinical diagnosis and treatment.

Hypertension is one of the independent risk factors for the development of cognitive dysfunction in patients with CSVD (23, 24), and the results of our study found that hypertension is an independent risk factor for the development of cognitive dysfunction in patients with CSVD. A prospective study showed that patients with CSVD combined with hypertension had a 1.5-fold increased risk of cognitive dysfunction compared with patients with normal blood pressure (25), and the result is the same as the results of most studies. Patients with CSVD combined with hypertension are prone to cognitive impairment probably because hypertension causes a series of oxidative stress and inflammatory processes, resulting in changes in neural, vascular and endothelial functions, and accelerates the process of pathological changes in CSVD, such as white matter hyper-signal, lacunar cerebral infarction, peripheral vascular gap enlargement, and cerebral microhemorrhage (23, 24, 26).

The total CSVD MRI score is a combination of four different MRI markers to indicate the degree of brain damage in CSVD patients (27). The results of our study found that the total CSVD MRI score was an independent risk factor for the development of cognitive dysfunction in patients with CSVD, and the total CSVD MRI score was positively associated with the development of cognitive dysfunction in patients with CSVD. When performing cognitive function tests, the heavier the total CSVD MRI load, the lower the score obtained by the patient. A prospective study by Jiang Y et al. (28) also showed that the total CSVD MRI load at the beginning of the study for participants included in the study was strongly associated with a decrease in MMSE scores at the end of follow-up, and that patients were at higher risk of developing dementia when more than three CSVD imaging markers were present at the beginning of the study. In addition, Li X et al. (29) showed that the total CSVD MRI score was an independent risk factor for cognitive dysfunction in the overall cognitive domain and more than three cognitive domains, and the results of our study are consistent with the findings of all these studies. The effect of The total CSVD MRI score on cognitive function may be due to its corresponding pathological changes that damage the structural and functional brain networks of patients (30), which in turn affects the functional integration capacity of structural brain networks and leads to cognitive dysfunction in CSVD patients (31).

Hcy is a sulfur-containing amino acid produced during methionine metabolism, which generates oxygen radicals with strong oxidative effects on vascular endothelial cells and is defined as a reactive vascular damage amino acid (32). High Hcy can cause endothelial dysfunction in the body and plays an important role in the pathogenesis of neurological disorders such as stroke and cognitive impairment. The results of our study found that high Hcy was an independent risk factor for the development of cognitive dysfunction in patients with CSVD, and previous studies have also shown that elevated Hcy is significantly associated with the development of cognitive dysfunction in patients (33). When the organism's Hcy is elevated, its induced oxidative stress, endothelial dysfunction, inflammation, and endoplasmic reticulum (ER) stress cause neurovascular dysfunction (34), which in turn puts CSVD patients at increased risk of cognitive dysfunction.

Years of schooling were considered to be significantly associated with the occurrence of cognitive dysfunction in patients with CSVD, and the results of our study showed that years of schooling were a protective factor against the occurrence of cognitive dysfunction in patients with CSVD, and the higher the level of schooling in patients with CSVD, the slower the decline in cognitive function. Most studies have shown that the higher the level of schooling of CSVD patients, the lower the odds of cognitive dysfunction (35, 36). Thus, low years of education are a risk factor for the development of cognitive dysfunction in patients with CSVD, and changes in cognitive function over time in patients are influenced by years of schooling (37, 38). In addition, higher levels of schooling are associated with greater neural reserve capacity formed in brain networks, which in turn promotes the development of compensatory neural circuits, ultimately improving resistance to inflammatory brain damage and delaying the onset of cognitive dysfunction (39, 40).

This study constructed a nomogram of predictive models based on four independent risk factors in patients with CSVD. Previous studies have performed nomogram construction based on CSVD patients, but no study has done a nomogram on the occurrence of cognitive dysfunction in patients with CSVD. In addition, the accuracy of the model was evaluated using calibration curves, ROC curves, and DCA curves in this study, and the results showed that the model has good accuracy and discriminatory ability, and has clinical validity, which can provide some reference for disease management of CSVD patients. However, our study also has some limitations. First, this study is a single-center retrospective study with limited sample size, and the results may have some inevitable biases. Second, this study did not conduct a stratified study on patients with different cognitive domains, and further research is needed to further explore. Third, the patients included in this study were those admitted to the hospital for treatment, and it is possible that the results of the study are not applicable to outpatients. Finally, the results of this study need to be further analyzed and validated by multicenter studies with larger sample sizes and prospective studies, to find more clinical predictors of cognitive dysfunction in CSVD patients and to provide greater clinical reference value for clinical intervention and delay the process of cognitive dysfunction in CSVD patients.

## Conclusion

In conclusion, hypertension, Hcy, total CSVD MRI Score, and years of schooling are independent risk factors affecting the development of cognitive dysfunction in patients with CSVD. Early clinical interventions targeting relevant independent risk factors are particularly important in the assessment and management of patients' conditions. Whether high years of schooling as a protective factor can be considered to delay cognitive decline by providing patients with relevant learning and memory exercises deserves further practice and demonstration by clinicians. Finally, it is expected that this study will provide some reference for clinicians in the assessment of CSVD patients' conditions and the development of individualized disease management strategies.

## Data availability statement

The raw data supporting the conclusions of this article will be made available by the authors, without undue reservation.

## Ethics statement

The studies involving human participants were reviewed and approved by the Medical Ethics Committee of Gansu Provincial Hospital. The patients/participants provided their written informed consent to participate in this study. Written informed consent was obtained from the individual(s) for the publication of any potentially identifiable images or data included in this article.

## Author contributions

LZ and FG contributed equally to this article, completed the study design, statistical analysis, and drafted the manuscript. YH and QS collect patient data from Hospital's Electronic Medical Record System. QZ and PH perform MoCA scale measurements for patients. YZ and YY performed imaging assessment and helped to embellish language. YZ conceived of the study and provided expert consultations and suggestions. All authors contributed to the study and approved the submitted version.

## Funding

This work was supported by the Gansu Province Health Industry Scientific Research Project (GSWSKY2020-03) and the Natural Science Foundation of Gansu Province (20JR5RA152).

## Conflict of interest

The authors declare that the research was conducted in the absence of any commercial or financial relationships that could be construed as a potential conflict of interest.

## Publisher's note

All claims expressed in this article are solely those of the authors and do not necessarily represent those of their affiliated organizations, or those of the publisher, the editors and the reviewers. Any product that may be evaluated in this article, or claim that may be made by its manufacturer, is not guaranteed or endorsed by the publisher.
